# Sirtuins: New Roles for Sirtuins in Aging and Disease

**DOI:** 10.1093/geroni/igaa057.2650

**Published:** 2020-12-16

**Authors:** Katrin Chua

## Abstract

Sirtuins are NAD+-dependent enzymes related to yeast silent information regulator-2, a histone deacetylase that prevents premature aging through chromatin silencing. Mammalian sirtuins, SIRT1-SIRT7, have key functions that protect against diverse aging-related pathologic states, from cancer to metabolic and neurodegenerative disease. In addition to chromatin regulation, mammalian sirtuins also govern myriad cellular processes through a growing list of enzymatic activities and substrates. This session will highlight new discoveries and directions in Sirtuin research.

